# Enhanced T-cell immunity and lower humoral responses following 5-dose SARS-CoV-2 vaccination in patients with inborn errors of immunity compared with healthy controls

**DOI:** 10.3389/fimmu.2025.1538453

**Published:** 2025-03-06

**Authors:** Vitor Gabriel Lopes da Silva, Gabriela Justamante Händel Schmitz, Kathleen E. Sullivan, Júlia Barbate, Maria Izabel de Haro Azinar, Carolina Sanchez Aranda, Maria Isabel de Moraes-Pinto

**Affiliations:** ^1^ Departamento de Pediatria, Escola Paulista de Medicina, Universidade Federal de São Paulo, São Paulo, Brazil; ^2^ Departamento de Clínica Médica, Universidade de São Paulo, São Paulo, SP, Brazil; ^3^ The Children’s Hospital of Philadelphia, Perelman School of Medicine, University of Pennsylvania, Philadelphia, PA, United States

**Keywords:** COVID-19 vaccines, booster, inborn errors of immunity, primary immunodeficiency disorders, SARS-CoV-2, microarray, immune response, ELISpot enzyme-linked immunospot

## Abstract

**Objective:**

Patients with Inborn Errors of Immunity (IEI) are at higher risk of severe SARS-CoV-2 infection. We evaluated humoral and cellular responses to COVID-19 vaccines in Brazilian patients with IEI and healthy controls.

**Methods:**

Fifty-five patients with IEI (13–61 years) and 60 controls (13–71 years) received inactivated SARS-CoV-2 (CoronaVac), non-replicating virus-vectored (ChAdOx1 nCoV-19, AstraZeneca) or monovalent mRNA (Original strain of BNT162b2, Pfizer-BioNTech) and bivalent mRNA (Original/Omicron BA.1, Pfizer-BioNTech) vaccines and were sampled five times. Diagnoses included common variable immunodeficiency (n=25), specific antibody deficiency (n=9), ataxia-telangiectasia (n=5), X-linked agammaglobulinemia (n=4), PIK3CD-related disorders (n=4), hyper-IgM syndrome (n=4), combined immunodeficiency (n=3), and STAT1 gain-of-function (n=1). Humoral immunity was assessed via multiplex microarray for Spike, Nucleocapsid, RBD-Wuhan, RBD-Delta, RBD-BA.1, RBD-BA.2 and RBD-BA.5 neutralizing antibodies. T-cell responses to Spike and Nucleocapsid were assessed using ELISpot.

**Results:**

Patients with IEI exhibited significantly lower levels of Nucleocapsid and RBD-neutralizing antibodies (p < 0.05). Notable differences in RBD-BA.2 (p = 0.008) and IgG-Nucleocapsid (p = 0.010) levels emerged over time. T-cell responses to Spike were stronger in patients with IEI post-booster (405 vs. 149 spot-forming cells/million PBMC; p = 0.002). Both groups showed enhanced Nucleocapsid-specific cellular responses over time (p = 0.017). COVID-19 hospitalization rates among patients with IEI with SARS-CoV-2 diagnosis dropped from 33.3% to zero after the first booster dose.

**Conclusions:**

While humoral responses to SARS-CoV-2 vaccines were weaker in patients with IEI, their cellular immunity was similar to controls. Boosters enhanced both humoral and cellular responses. After completion of the vaccination protocol, none of the patients with IEI were hospitalized with COVID-19. Robust T-cell responses may play a critical role in protecting patients with IEI from severe COVID-19 and mortality.

## 1 Introduction

The COVID-19 pandemic has affected more than 777 million people and killed more than 7.1 million people worldwide as of January 2025. In Brazil alone, there were nearly 38 million confirmed cases and 703,000 deaths in the same period (1). Specific conditions, such as combined immunodeficiencies, immune dysregulation disorders [especially defects in tolerance, such as IPEX (immune dysregulation, polyendocrinopathy, and enteropathy X-linked syndrome), and other “TRegopathies”], and defects in the type I interferon pathway are associated with worse COVID-19 outcomes (2).

Although patients with Inborn Errors of Immunity (IEI) are at increased risk of developing severe COVID-19 (2, 3), they can develop potentially protective immune responses following vaccination, which can be further enhanced by booster doses. The wide range of vaccination response rates may be attributed to different vaccination protocols (4, 5), different underlying conditions and the small sample sizes of published studies (6). Antibody responses alone may not necessarily be correlated with the prevention of COVID-19 hospitalization, as other immunological mediators, such as vaccine-specific T-cell responses, can prevent or reduce the severity of COVID-19 (7–10).

Published studies of responses after two doses of COVID-19 vaccination in patients with IEI indicated that 48.5% to 86.0% of patients developed neutralizing antibodies against SARS-CoV-2 (4, 5, 11–15), whereas 73.1% to 87.0% of patients exhibited T-cell responses (4, 5, 11–13, 15). The COVID-19 vaccines induced substantially lower immune responses in patients with IEI than in healthy controls (4, 11, 12, 16). These differences were especially significant concerning neutralizing antibodies to Omicron variants with relevant specific mutations that induce an immune escape (17).

In Brazil, the vaccination of patients with IEI started in May 2021 after more than 400,000 deaths. Four months later, in September 2021, the administration of the third COVID-19 vaccine dose for immunosuppressed individuals began. The immunization was preferably performed with an original strain of BNT162b2 (Pfizer-BioNTech) or, alternatively, with a viral vector vaccine of Ad26.COV2.S (Janssen) or ChAdOx1 nCoV-19 (AstraZeneca) (18). The administration of bivalent mRNA (Original/Omicron BA.1, Pfizer-BioNTech) vaccine as booster began in March 2023.

The high burden of COVID-19 in Brazil led us to analyze responses in patients with IEI followed at the Immunology Clinic at the Federal University of Sao Paulo. This patient population is vulnerable and the level of protection has not yet been characterized. We concluded that most patients with IEI respond to COVID-19 immunization with a three-dose primary vaccination schedule followed by two booster doses (4th and 5th vaccines doses) although humoral and T cell responses differed.

## 2 Methods

### 2.1 Ethics statement

This study adheres to the principles of the Declaration of Helsinki and was approved by the Brazilian National Research Ethics Committee (number 51535921.2.0000.5505). All participants provided written informed consent before enrollment.

### 2.2 Study design

**Figure 1:**
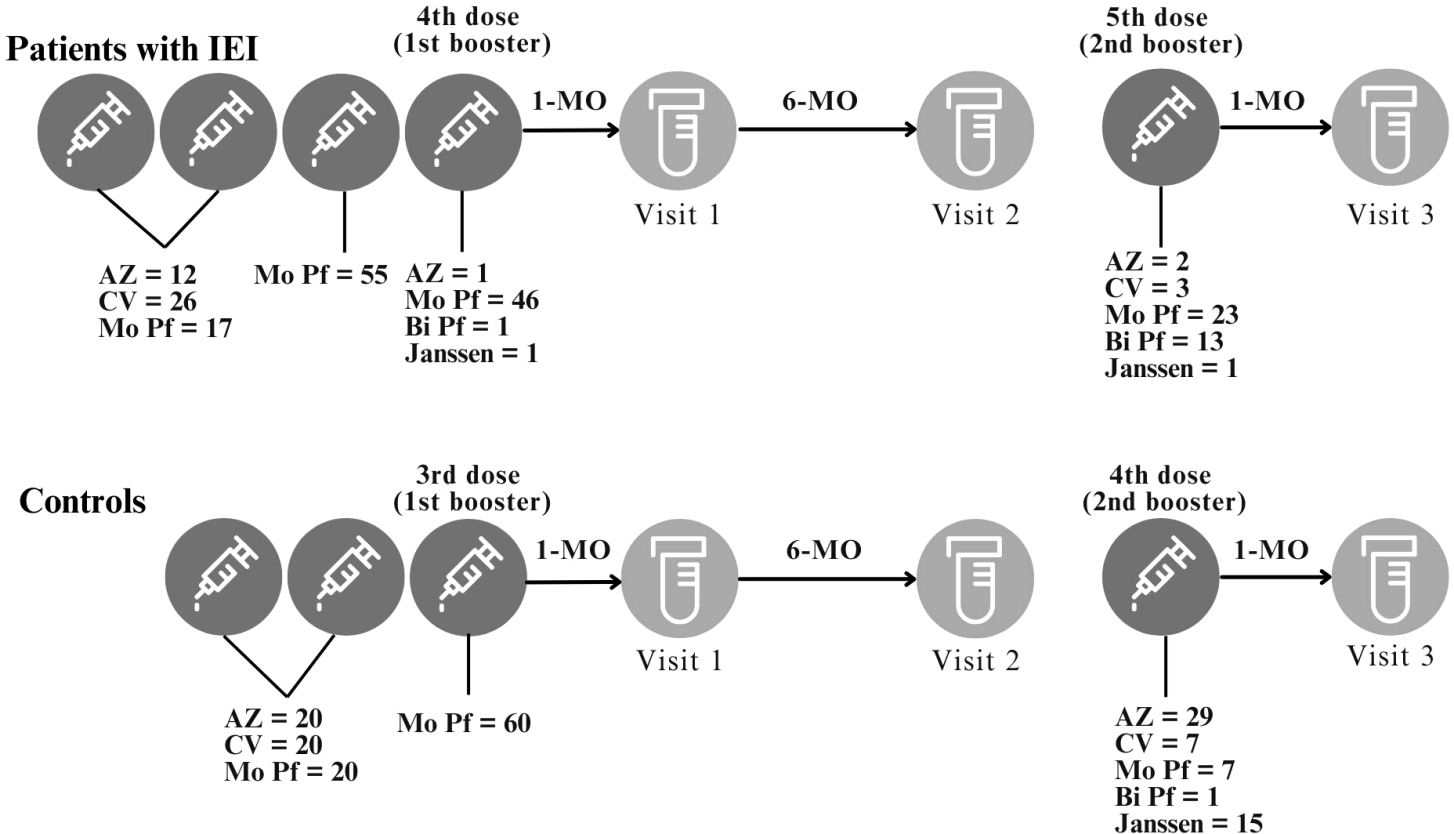
Study design. Study visits and intervals between study visits or between vaccination and study visits are depicted. The BNT162b2 (*Mo Pf*) vaccine was used as the third vaccine dose. The 4^th^ and 5^th^ vaccine doses were based on local availability. For the control cohort, the 3^rd^ dose is the first booster and the 4^th^ dose is the second booster. For the IEI cohort, the 4^th^ dose is the first booster and the 5^th^ dose is the second booster. This study compared the immune response after the 1^st^ and 2^nd^ booster for both IEI and Control cohorts. AZ = ChAdOx1 nCoV-19, AstraZeneca; CV = CoronaVac; Mo Pf = monovalent mRNA original strain of BNT162b2, Pfizer-BioNTech; Bi Pf = bivalent mRNA Original/Omicron BA.1, Pfizer-BioNTech; Janssen = Ad26.COV2.S.

This was a prospective cohort study that took place between October 2021 and November 2023 (**Supplementary Figure S1** in the **Supplementary Material**). Patients with an established IEI diagnosis from a Brazilian reference center, the Immunology Clinic of the Federal University of São Paulo, who received three doses of COVID-19 vaccines, were offered the opportunity to join the study. A control group of healthy individuals of similar sex and age distributions were also invited to participate. One-third of patients with IEI and controls had a basic immunization schedule that consisted of two doses of either CoronaVac, BNT162b2 (Pfizer-BioNTech) or ChAdOx1 nCoV-19 (AstraZeneca), followed by a third dose of BNT162b2 (Pfizer-BioNTech). The fourth and fifth doses varied according to the availability of the different COVID-19 vaccine platforms in both the IEI cohort and the control cohort. Patients with IEI had blood samples collected 1 and 6 months after the fourth COVID-19 vaccine dose (the first booster for the IEI cohort) and 1 month after the fifth COVID-19 vaccine dose (the second booster for the IEI cohort). Blood samples were collected from control individuals 1 and 6 months after the third COVID-19 vaccine dose (the first booster for the control cohort) and 1 month after the fourth COVID-19 vaccine dose (the second booster for the control cohort) (**Figure 1**).

### 2.3 Flow cytometry immunophenotyping

Lymphocyte subsets (CD3^+^ T, CD4^+^ T, CD8^+^ T, CD19^+^ and NK cells) were assessed via a single platform (TruCount and Multitest, BD Biosciences, San Jose, CA) on a BD FACSCalibur™ 4-color flow cytometer using CellQuest software (BD Biosciences, San Jose, CA). Data analysis was performed via MultiSET v3.0.2 software (Becton, Dickinson and Company, New Jersey, USA).

### 2.4 PBMC isolation

Peripheral blood mononuclear cells (PBMCs) were isolated using Ficoll gradient density. Following isolation, the cells were stored in liquid nitrogen for later ELISpot assays to evaluate peptide-induced cytokine production.

### 2.5 Evaluation of the humoral response using SARS-CoV-2 NTChip^®^ assay

Serum samples were separated on the day of blood collection and stored at -80°C for later analysis. The humoral immune response was assessed via the commercial SARS-CoV-2 NTChip^®^ Test (V-NTCGOK) (Viramed Biotech AG, Germany). This is an *in vitro* qualitative and quantitative multiplex microarray microsystem based on an enzyme immunoassay previously validated against the 50% Plaque Reduction Neutralization Test (PRNT_50_) to determine the level of neutralizing antibodies against purified specific surface antigens from the RBD portion of the Spike protein, in printed spot triplets in a nitrocellulose membrane, from variants and subvariants of SARS-CoV-2, such as Wuhan – wild type, Delta, Omicron BA.1 – B.1.1.529 and BA.2 and BA.5, which are on each well of a 96-well plate (**Supplementary Figure S2**).

During the development of the NTChip^®^ (19), in order to validate the PRNT_50_ – the gold standard to determine the level of neutralizing antibodies for many viral diseases – six groups of sera were evaluated: 1) pre-pandemic (W-O-), 2) previous Omicron variant (W+O-), 3) infected/vaccinated post Omicron (W+O+), 4) naïve Omicron positive (W-O+), 5) Omicron positive 0-2 days and 6) 10 days after infection. Sensitivity and specificity results for RBD-Wuhan, RBD-Omicron BA.1 and Nucleocapsid (N) were: 96.7% and 100.0%; 95.7% and 99.1%, and 95.1% and 94.0%, respectively.

To detect neutralizing antibodies, the assay uses the cellular receptor angiotensin conversion enzyme-2 (ACE-2). Results can be expressed for neutralizing antibodies in percentage of inhibition, which is transformed in IU/mL by the ViraChip^®^ Software after the results are read by the ViraChip^®^ Scanner. The NTChip^®^ test can also assess a previous contact with the virus using Nucleocapsid (N) printed in spots triplets on the nitrocellulose; IgG antibodies from the sera are detected using a conjugated anti-IgG.

The result for captured IgG can be expressed in arbitrary units (AU) and in binding arbitrary units (BAU). For quantitative results, the NTChip^®^ was calibrated against the WHO international standard NIBSC 21/338 (20). The quantitative cutoffs (NIBSC 21/338) for Wuhan, Omicron (BA.1) and Nucleocapsid were: 9.1 IU/mL, 626.5 IU/mL and 16.4 BAU/mL, respectively, which can be translated into the following percentages of inhibition: 9%, 29% and 74 AU, respectively. As only RBD-Wuhan, RBD-BA.1 and Nucleocapsid were validated against PRNT_50_, there are no cutoffs for RBD-Delta, RBD-BA.2 and RBD-BA.5.

### 2.6 Evaluation of the cellular response using ELISpot assay

To evaluate the specific T-cell immune response to SARS-CoV-2, the commercial T-SPOT^®^COVID kit (Oxford Immunotec, Oxford, England) was used. This standardized method detects CD4^+^ T and CD8^+^ T cells that secrete interferon-gamma (IFN-γ) in response to stimulation with antigens via two specific peptide pools from SARS-CoV-2, one from the Spike protein and the other from the Nucleocapsid. Each assay was read using the AID EliSpot fluorescence microplate reader (Autoimmun Diagnostika GMBH, Germany) and AID EliSpot 7.0 software. The test result is considered positive if either Spike and/or Nucleocapsid have a count of 8 spots or more per 250,000 PBMC (**Supplementary Figure S3**). The test result is considered negative if both have a count of 4 spots or less. Results of 5, 6, or 7 spots are considered indeterminate according to the manufacturer. A reactive result indicates the presence of SARS-CoV-2-sensitized effector T cells in the sample. A non-reactive result indicates that SARS-CoV-2-sensitized effector T cells were not detected in the sample.

### 2.7 Statistical analysis

Associations between two categorical variables were assessed using the Chi-square test or Fisher’s Exact test. Comparisons of means between two groups were performed using Student’s t-test. Normality assumption was checked using Kolmogorov-Smirnov test. In case of violation of this assumption, the nonparametric Mann−Whitney test was used alternatively.

Geometric means (GMT) and 95% confidence intervals (CIs) are presented for antibody assessment. Comparisons of variable levels by group and time were conducted via linear models with random effects (21) on log-transformed variables. This model assessed the effects of three components: time, group, and interaction between group and time. The presence of interaction between group and time indicates that group means evolve differently over time.

For qualitative cellular responses, logistic regression models with random effects were used. The linear model with random effects assumes data normality. However, deviation from normality does not bias estimates (22).

A significance level of 5% was used for all the statistical tests. Analyses were performed using SPSS 20.0 and STATA 17.

All the graphs were generated via GraphPad Prism 10.4.3.

## 3 Results

### 3.1 Baseline characteristics

A total of 55 patients with IEI and 60 controls were included in the study. The two groups were similar with respect to sex and age (p >0.05). The median age of patients with IEI was 27.3 years (ranging from 13.3 to 61.9 years) and that of the controls was 25.0 years (13.5 to 71.2 years) (p = 0.991). Patients with IEI patients had a median BMI of 21.91 kg/m² (15.15 to 36.51 kg/m²), which was lower than that of the controls (p = 0.026). The groups were comparable regarding the following comorbidities: systemic arterial hypertension, diabetes mellitus, heart disease, and obesity. Among the patients with IEI, 40% (22/55) had pulmonary diseases (asthma, bronchiectasis, pulmonary fibrosis, and lobectomy) and 10.9% (6/55) had other diseases, such as nephropathy, hepatopathy, neuropathy, and myasthenia gravis. Due to the exclusion criteria for the control group, no individuals in this group had diabetes mellitus, heart disease, pulmonary disease, or other comorbidities (**Table 1**).

**Table 1:** Clinical and demographic data of the study participants.

	IEI cohort (n=55)	Control cohort (n=60)	*p*
**Male sex, n (%)**	29 (52.7)	29 (48.3)	0.638^a^
**Age (years) median (min–max) **	27.3 (13.3-61.9)	25.0 (13.5-71.2)	0.991^d^
**Age group, n (%)** 12 to 19 years	13 (23.6)	4 (6.7)	
20 to 44 years	31 (56.4)	48 (80.0)	
45 to 59 years	9 (16.4)	4 (6.7)	
≥ 60 years	2 (3.6)	4 (6.7)	
**BMI (kg/m²) median (min–max)**	21.91 (15.15-36.51)	24.30 (17.36-40.65)	**0.026^c^**
Comorbidities, n (%)			
Systemic Arterial Hypertension	6 (10.9)	3 (5.0)	0.307^b^
Diabetes Mellitus	2 (3.6)	0 (0.0)	0.227^b^
Pulmonary diseases*	22 (40.0)	0 (0.0)	**<0.001^a^**
Heart diseases#	3 (5.5)	0 (0.0)	0.106^b^
Obesity (BMI > 29.9 kg/m²)	4 (7.3)	5 (8.3)	1.000^b^
Lymphocyte Subpopulations (cells/mm³) median (min–max)			
CD45^+^	1989 (307-10630)	2168 (793-4662)	**0.050^d^**
CD3^+^ T	1424 (269-7995)	1655 (524-3735)	**0.045^d^**
CD4^+^ T	664 (102-3884)	960 (348-2727)	**<0.001^d^**
CD8^+^ T	615 (79-3938)	593 (182-1473)	0.996^d^
CD19^+^ (B Lymphocytes)	174 (0-1283)	269 (101-982)	**0.005^d^**
CD16^+^CD56^+^ (NK cells)	245 (38-1228)	266 (83-1467)	0.195^d^

n, number of individuals; min, minimum; max, maximum; BMI, Body Mass Index. *Pulmonary diseases: asthma, bronchiectasis, pulmonary fibrosis, and lobectomy; #Heart diseases: arrhythmia and cardiopathy; p: descriptive level of the Chi-Square test (a), Fisher’s Exact test (b), Student’s t-test (c), and Mann−Whitney test (d).

The bold value represents statistically significant results.

The median numbers of CD3^+^ T lymphocytes (p = 0.045), CD4^+^ T lymphocytes (p < 0.001), and B lymphocytes (p = 0.005) were lower in the IEI group than in the control group. No differences were observed for CD8^+^ T lymphocytes or NK cells (p = 0.996 and p = 0.195, respectively) (**Table 1**).

All controls (n = 60) and 42 patients with IEI completed all 5 study visits (**Figure 1**). Among the 13 individuals who did not complete the visits, 6 did not want to receive the 4th vaccine dose, and 6 did not want to receive the 5th vaccine dose. One patient with X-linked Agammaglobulinemia had a history of pyoderma gangrenosum in the left lower limb that worsened during the study period; owing to frequent and long periods of hospitalization, he could not receive the 5th vaccine dose; this event was not considered to be caused by the vaccination.

The distribution of patients with IEI according to the first two COVID-19 vaccine doses administered is depicted in **Table 2**. Immunoglobulin replacement therapy (IGRT) was used by 48/55 (87.3%) patients with IEI. No differences were noted between the IEI and control groups with respect to the distribution of the types of the first two vaccine doses (p = 0.245). For the first and second doses, 47.3% (26/55) of the patients received CoronaVac, 30.9% (17/55) received BNT162b2 (Pfizer-BioNTech), and 21.8% (12/55) received ChAdOx1 nCoV-19 (AstraZeneca). The controls had an equal distribution of 33.3% for each vaccine (**Figure 1**).

**Table 2:** Characteristics of the patients with IEI.

	IEI cohort (n=55)	IEI ChAdOx1 nCoV-19, AstraZeneca (n=12)	IEI CoronaVac (n=26)	IEI BNT162b2, Pfizer-BioNTech (n=17)
Diagnosis of IEI, n (%)				
Common Variable Immunodeficiency (CVID)	25 (45.4)	5 (41.7)	15 (57.7)	5 (29.4)
Specific Antibody Deficiency	9 (16.4)	3 (25.0)	3 (11.5)	3 (17.6)
Ataxia-Telangiectasia	5 (9.1)	2 (16.7)	1 (3.8)	2 (11.8)
X-Linked Agammaglobulinemia	4 (7.3)	1 (8.3)	2 (7.7)	1 (5.9)
Hyper-IgM Syndrome	4 (7.3)	–	3 (11.5)	1 (5.9)
PIK3CD Mutation (APDS 1)	4 (7.3)	1 (8.3)	–	3 (17.6)
Combined Immunodeficiency	3 (5.4)	–	2 (7.7)	1 (5.9)
STAT-1 GOF	1 (1.7)	–	–	1 (5.9)
**Use of IVIg, n (%)**	48 (87.3)	10 (83.3)	22 (84.6)	16 (94.1)
**Age (years) median (min–max) **	27.3 (13.3-61.9)	33.6 (19.5-60.6)	30.0 (19.0-53.3)	17.0 (13.3-61.9)
**Age at Diagnosis (years)** **median (min–max) **	14 (3-47)	27 (4-47)	18 (3-37)	8 (4-43)

n, number of individuals; min, minimum; max, maximum; IVIg, intravenous human immunoglobulin.

The bold value represents statistically significant results.

In both groups, all participants received original strain of BNT162b2 (Pfizer-BioNTech) as the third vaccine dose, according to the study protocol. For the fourth dose, following vaccination campaign priorities, patients with IEI predominantly received original strain of BNT162b2 (93.9%; 46/49). One patient received ChAdOx1 nCoV-19 (AstraZeneca), one received Ad26.COV2.S (Janssen), and one received bivalent mRNA (Original/Omicron BA.1, Pfizer-BioNTech) vaccine. Among controls, the fourth dose was predominantly with ChAdOx1 nCoV-19 (AstraZeneca) (49.2%; 29/59), followed by Ad26.COV2.S (Janssen) (25.4%; 15/59), CoronaVac, and original strain of BNT162b2 (11.9% each; 7/59), with one person receiving bivalent mRNA (Original/Omicron BA.1, Pfizer-BioNTech) (1.7%) (**Figure 1**). One control did not receive the fourth dose as he was under 18 years of age, and this vaccine was not yet available for that age group during the study period. Since patients with IEI were prioritized in the vaccination campaign, the mean interval between the third and fourth doses was smaller among patients with IEI compared to controls (150.2 days vs. 234.8 days; p < 0.001, Mann-Whitney test). This also occurred between the second and third doses: 95.5 days in patients with IEI and 174.3 days in controls (p < 0.001, Student’s t test).

Patients with IEI predominantly received the fifth dose (second booster) with original strain of BNT162b2 (54.8%; 23/42), followed by bivalent mRNA (Original/Omicron BA.1, Pfizer-BioNTech) (31%; 13/42). Three patients received CoronaVac (7.1%), two received ChAdOx1 nCoV-19 (AstraZeneca) (4.8%), and one received Ad26.COV2.S (Janssen) (2.4%) (**Figure 1**). As the bivalent mRNA (Original/Omicron BA.1, Pfizer-BioNTech) vaccine was not yet available at the start of the second booster campaign for patients with IEI, some individuals did not receive it. The mean interval between the fourth and fifth doses was 247 days, ranging from 123 to 514 days.

### 3.2 Humoral response assessed by RBD-Wuhan, RBD-Delta, RBD-BA.1, RBD-BA.2 and RBD-BA.5 neutralizing antibodies and anti-Nucleocapsid total IgG

**Figure 2:**
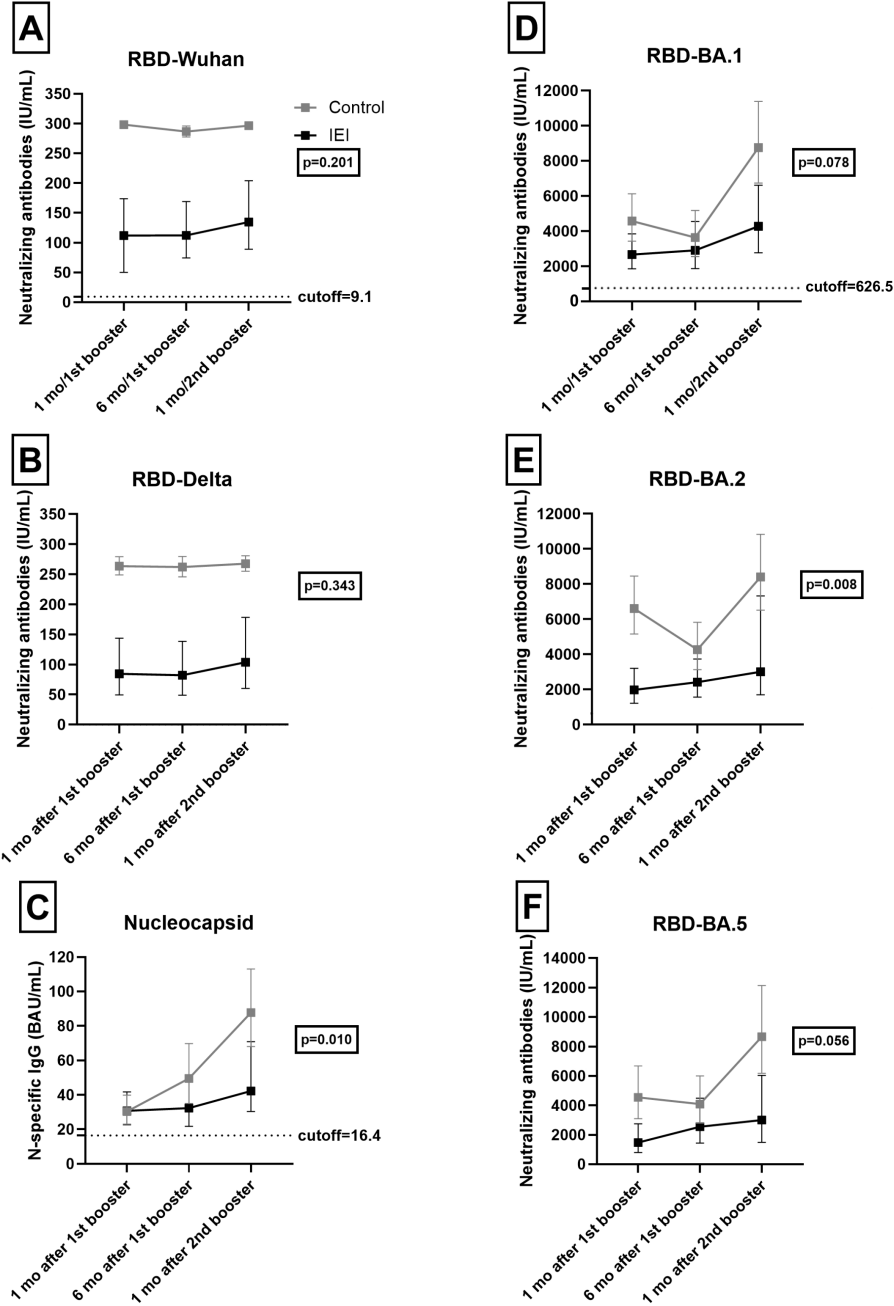
Humoral response. RBD-Wuhan **(A)**, RBD-Delta **(B)**, Nucleocapsid N-specific IgG **(C)**, RBD-BA.1 **(D)**, RBD-BA.2 **(E)** and RBD-BA.5 **(F)** antibodies 1 and 6 months after the 1^st^ booster and 1 month after the 2^nd^ booster in IEI and Control. For the control cohort, the 3^rd^ dose is the first booster and the 4^th^ dose is the second booster. For the IEI cohort, the 4^th^ dose is the first booster and the 5^th^ dose is the second booster. GMT values and 95% Confidence Intervals are shown. The dotted horizontal line is the quantitative cut-offs (NIBSC 21/338) for RBD-Wuhan (9.1 IU/mL), RBD-BA.1 (626.5 IU/mL) and Nucleocapsid (16.4 BAU/mL). No cut-offs are available for RBD-Delta, RBD-BA.2 and RBD-BA.5.

We assessed neutralizing antibodies to RBD-BA.1, RBD-BA.2, and RBD-BA.5 recognizing that there is some cross-reactivity and some antibodies related to natural infection (23, 24) (**Supplementary Figure S1**). We noted lower levels of neutralizing antibodies at first assessment for most epitopes tested. Changes in the GMTs of antibodies over time were evaluated via a linear regression model with random effects (**Figure 2**; see **Supplementary Table S1** for seropositivity data and **Supplementary Table S2** in the **Supplementary Material** for detailed data). Compared with controls, lower antibody levels for all variables analyzed were observed in patients with IEI (p < 0.05).

The rate of change of antibodies to RBD-Wuhan (p = 0.201) and RBD-Delta (p = 0.343) was similar over time when patients with IEI and controls were compared. Close to significant differences were also observed for antibodies to RBD-BA.1 (p = 0.078) and RBD-BA.5 (p = 0.056) variants. Over time, the two cohorts also showed a significantly distinct response to RBD-BA.2 neutralizing antibodies (p = 0.008). IgG-Nucleocapsid antibodies clearly differed over time with less increase over time apparent in patients with IEI (p = 0.010).

For RBD-BA.2, controls had lower GMT antibodies 6 months after the 1st booster (4266 IU/mL; 95% CI, 3124-5827), but antibodies increased 1 month after the 2nd booster (8402 IU/mL; 95% CI, 6513-10838), as did levels 1 month after the 1st booster (6603 IU/mL; 95% CI, 5159-8451). In the IEI cohort, the GMT was greater only 1 month after the 2nd booster (3002 IU/mL; 95% CI 1690-5329) than it was 1 month after the 1st booster (1975 IU/mL; 95% CI 1217-3203).

For Nucleocapsid, controls had higher GMT antibodies after the 2nd booster (87.8 BAU/mL; 95% CI, 68.1-113.1; p < 0.001) than 1 month and 6 months after the 1st booster, with similar GMTs between these time points [30.4 BAU/mL (95% CI, 23.1-39.9) and 49.5 BAU/mL (95% CI, 35.0-69.8), respectively]. In contrast, patients with IEI had similar GMTs across all evaluated time points [30.7 (95% CI, 22.6-41.7), 32.4 (95% CI, 21.8-48.3) and 42.2 BAU/mL (95% CI, 30.3-58.9)].

The RBD-BA.1, RBD-BA.2, RBD-BA.5 and Nucleocapsid antibodies were significantly increased 1 month after the second booster. For Nucleocapsid, IEI and control cohorts had similar GMTs 1 month after the 1st booster (30.7 vs 30.4 BAU/mL); however, 6 months after the 1st booster and 1 month after the 2nd booster, the IEI cohort had significantly lower GMTs than the controls did (32.4 vs 49.5 BAU/mL and 42.2 vs 87.8 BAU/mL, respectively). No time effect was observed for RBD-Wuhan (p = 0.173) or RBD-Delta antibodies (p = 0.376).

No significant differences were observed in the humoral response between IEIs and controls who received CoronaVac, ChAdOx1 nCoV-19 (AstraZeneca), or BNT162b2 (Pfizer-BioNTech) as their first two doses of COVID-19 vaccines (**Supplementary Table S3** in the **Supplementary Material**).

### 3.3 T-cell response to Spike and Nucleocapsid SARS-CoV-2

**Figure 3:**
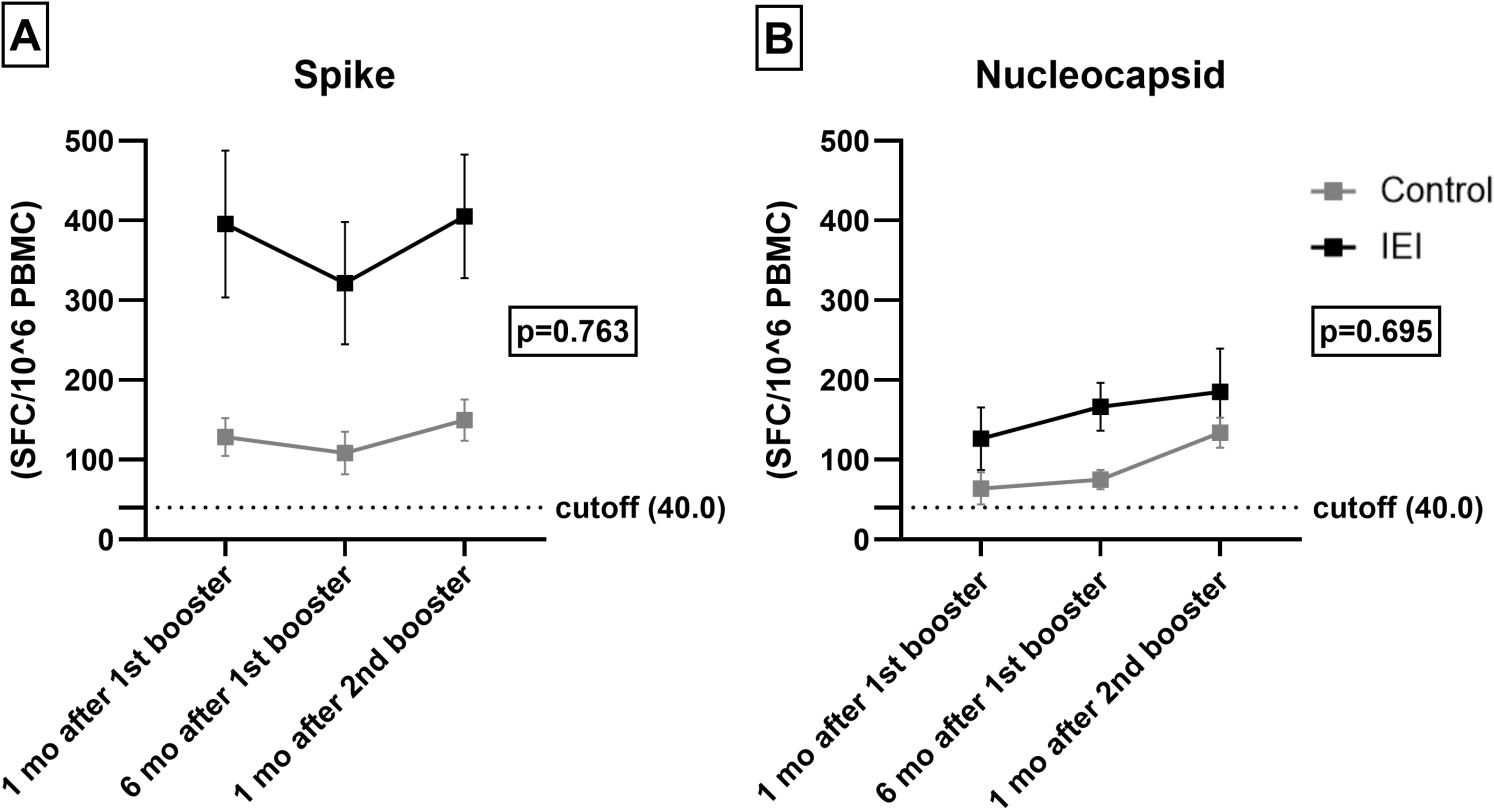
T-cell response. Spike **(A)** and Nucleocapsid **(B)** specific T-cell 1 and 6 months after the 1^st^ booster and 1 month after the 2^nd^ booster in IEI and Control. For the control cohort, the 3^rd^ dose is the first booster and the 4^th^ dose is the second booster. For the IEI cohort, the 4^th^ dose is the first booster and the 5^th^ dose is the second booster. Mean values of SFC/10^6^ PBMC with Errors bars are shown. The dotted horizontal line is the responder cut-off of the T-SPOT COVID assay (40 SFC/10^6^ PBMCs).

T cell and humoral responses may be discordant and T cell responses are thought to offer some level of protection in COVID-19. We therefore assessed T cell responses in patients and controls. A similar evolution of cellular response to Nucleocapsid (p = 0.763) and Spike (p = 0.695) was observed over time for IEI and control cohorts (**Figure 3**, **Supplementary Table S4** in the **Supplementary Material**). The IEI cohort presented a greater T-cell response to Spike in terms of the mean number of spots per million (SFC/10^6^) PBMCs than did the control cohort (p = 0.002), but a similar Nucleocapsid response (p = 0.180).

Within group analysis of change related to time revealed that the 1 and 6 months post-1st booster responses were lower than those at one month post-2nd booster (p = 0.017) for Nucleocapsid. This pattern was not observed for Spike (p = 0.445).

The vaccines received in the first two doses (CoronaVac, ChAdOx1 nCoV-19 or BNT162b2) did not affect the mean number of spots for Nucleocapsid (p = 0.554) and Spike (p = 0.554) (**Supplementary Table S5** in the **Supplementary Material**).

### 3.4 COVID-19 and hospitalizations

The goal of vaccination is to reduce mortality and hospitalizations. We therefore assessed the severity of infection among vaccinated individuals. Among the 115 participants, 71 were found to have COVID-19 before and during the study, as assessed by SARS-CoV-2 RT−PCR or antigen test detection.

**Figure 4:**
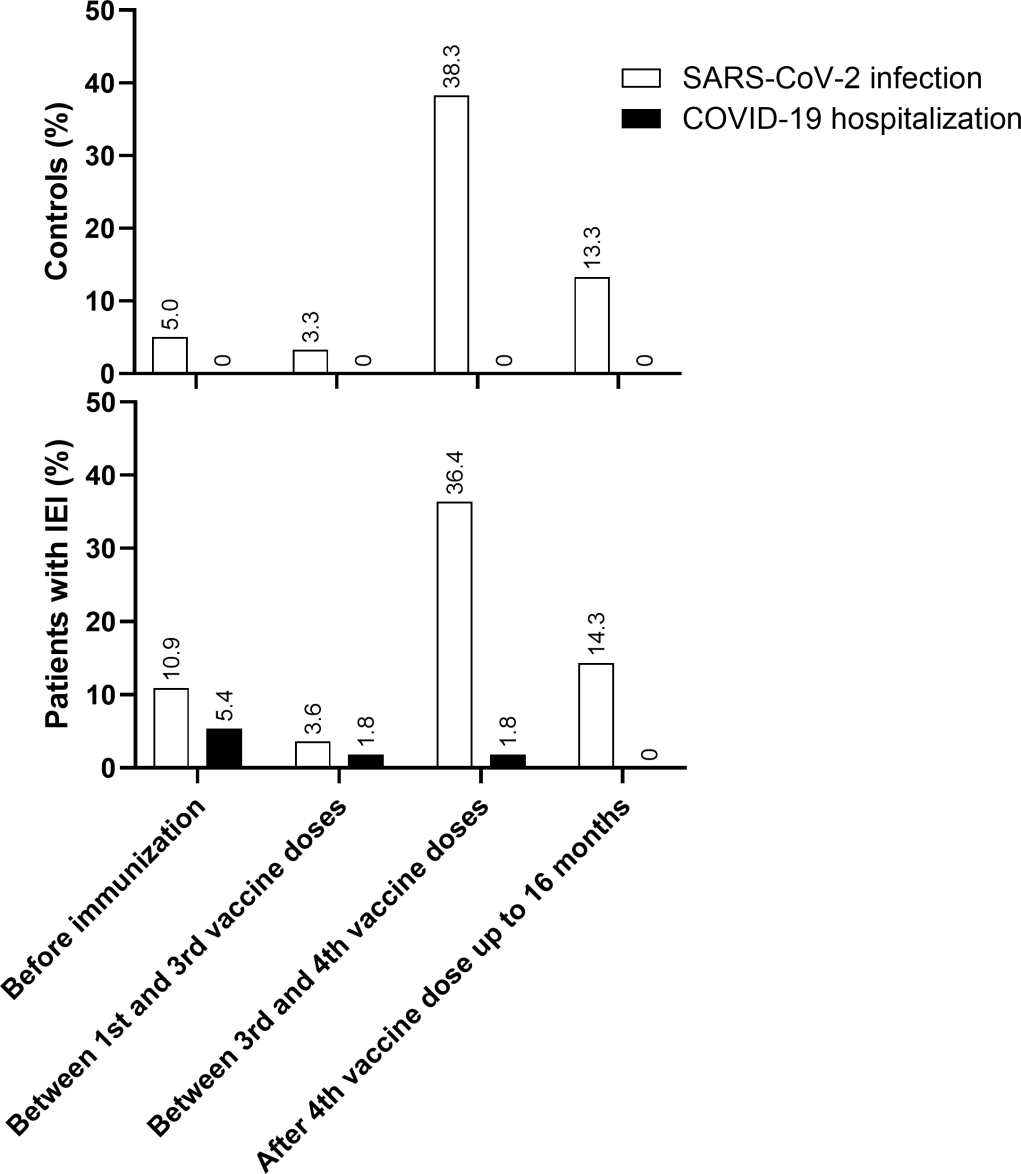
COVID-19 and hospitalizations. SARS-CoV-2 infection and COVID-19 hospitalization in Control and IEI cohorts before immunization, between 1^st^ and 3^rd^ vaccine dose, between 3^rd^ and 4^th^ vaccine dose and after 4^th^ vaccine dose up to 16 months of follow-up. For the control cohort, the 3^rd^ dose is the first booster and the 4^th^ dose is the second booster. For the IEI cohort, the 4^th^ dose is the first booster and the 5^th^ dose is the second booster.

Before immunization, 9/55 (16.3%) of patients with IEI had confirmed COVID-19: 6/55 (10.9%) did not need hospitalization and 3/55 (5.4%) required hospitalization. Between the 1st and 3rd vaccine dose, 3/55 (5.4%) had confirmed COVID-19, of which 2/55 (3.6%) did not need hospitalization and 1/55 (1.8%) had to be hospitalized. Between the 3rd vaccine dose and the 4th dose, during the Omicron circulation, 21/55 (38.2%) developed SARS-CoV-2 infection and only 1/55 (1.8%) was admitted to hospital. After the 4th dose and up to 16 months, 7/49 (14.3%) had confirmed SARS-CoV-2 infection, but none had to be hospitalized (**Figure 4**).

In the control group, 3/60 (5.0%) individuals developed SARS-CoV-2 infection before immunization and 2/60 (3.3%) between the 1st and 3rd vaccine dose; 23/60 (38.3%) were infected between the 3rd dose and the 4th dose, and 8/60 (13.3%) were infected after that up to 16 months of follow-up. None of them needed hospitalization (**Figure 4**).

The incidence of COVID-19 diagnosis was greater after the 3rd dose, when the Omicron variant was circulating in Brazil (**Supplementary Figure S1** in the **Supplementary Material**). However, although 77.1% (27/35) of the patients with IEI and 86.1% (31/36) of the controls were diagnosed with COVID-19 after the 3rd vaccine dose, all cases were mild, except for one patient with IEI who still required hospitalization but did not need oxygen therapy or ICU admission. Thus, vaccination achieved its overall clinical goal in our IEI cohort.

As there were only 5 patients who were hospitalized in this cohort, with 2 occurring after the start of the study, it is not possible to analyze specific details of the humoral or cellular responses that might have led to a higher probability of hospitalization compared to other patients. However, a qualitative analysis was performed.

After the start of the study, one 38-year-old female patient with CVID, who had a confirmed SARS-CoV-2 infection 12 days after her first dose and very likely without antibodies in her IVIg (June 2021) required oxygen therapy and ICU admission. She had a mild reinfection 8 months later. At study admission (one month after the 3rd vaccine dose, CoronaVac/CoronaVac/BNT162b2 scheme), her immunological status was CD4^+^ T = 664 cells/mm^3^, CD8^+^ T = 849 cells/mm^3^, CD19^+^ = 213 cells/mm^3^, with neutralizing antibodies detected against all SARS-CoV-2 variants (e.g., NAb anti-RBD-BA.5 = 20,925.0 IU/mL) and a robust cellular response (Nucleocapsid T-cell response = 53 SFC/10^6^ PBMC, Spike T-cell response = 367 SFC/10^6^ PBMC).

The other hospitalized patient was a 29-year-old male with AT, who tested positive for SARS-CoV-2 RT-PCR in June 2022, 153 days after receiving his 3rd vaccine dose. Differently from the other patient, he did not require oxygen therapy or ICU admission. At admission to study (one month after the 3rd vaccine dose, ChAdOx1 nCoV-19/ChAdOx1 nCoV-19/BNT162b2 scheme, and 4 months before COVID-19 hospitalization), he had CD4^+^ T = 202 cells/mm^3^, CD8^+^ T = 79 cells/mm^3^, CD19^+^ = 6 cells/mm^3^. He developed a robust cellular response (Spike T-cell response = 163 SFC/10^6^ PBMC), despite lymphopenia and was receiving IVIg, likely containing antibodies (as he had low B cell numbers, albeit lower antibody levels compared to other patients, e.g., NAb anti-RBD-BA.5 = 79.7 IU/mL). He was infected 4 months after vaccination during Omicron circulation (June 2022). Hospitalization was mainly for medical observation, without need for oxygen therapy or ICU admission.

### 3.5 Correlation between humoral and cellular responses

**Figure 5:**
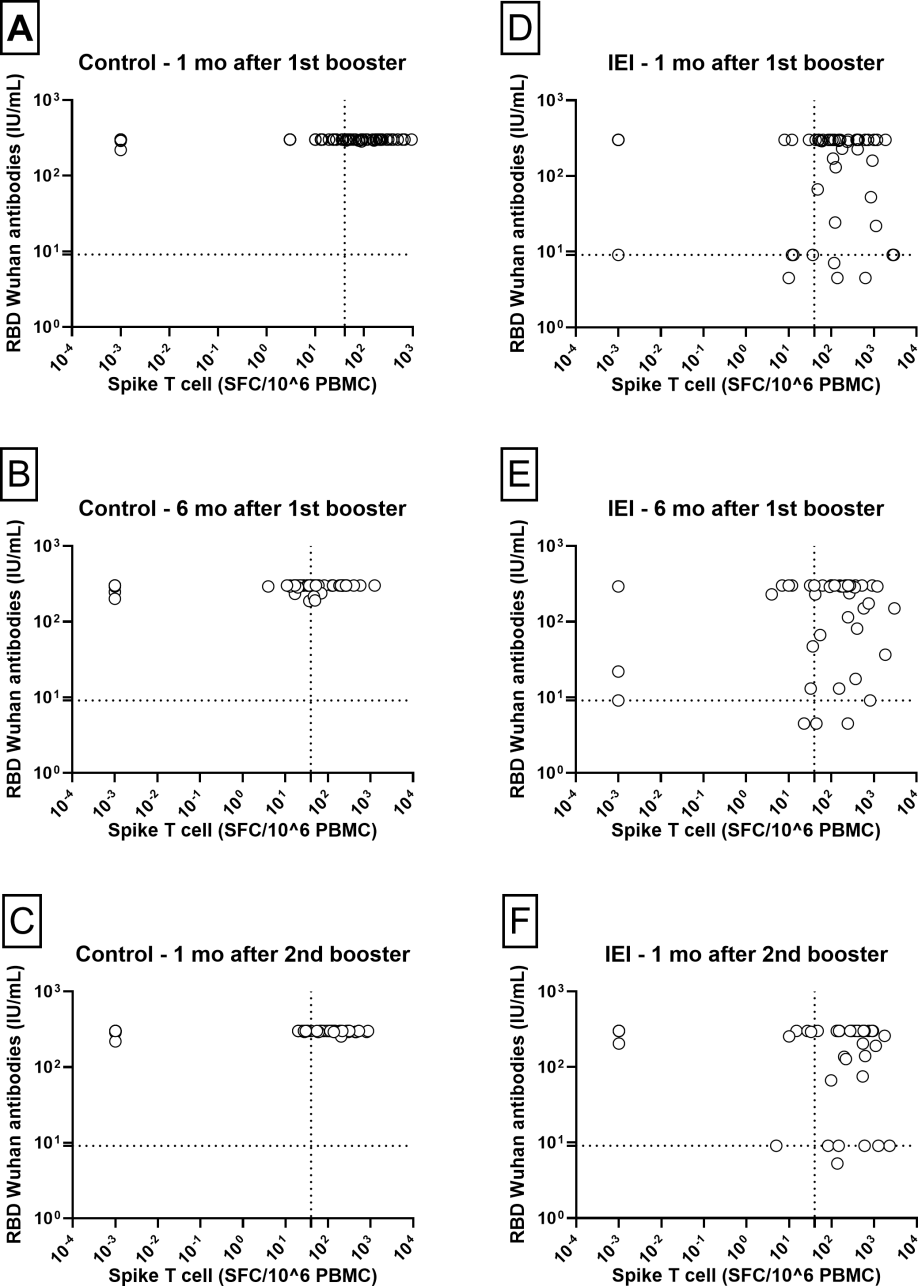
Humoral vs T-cell responses. Correlation between NAb RBD-Wuhan and Spike T-cell responses 1 and 6 months after the 1^st^ booster and 1 month after the 2^nd^ booster in Control [**(A–C)** respectively] and IEI [**(D–F)** respectively] cohorts. For the control cohort, the 3^rd^ dose is the first booster and the 4^th^ dose is the second booster. For the IEI cohort, the 4^th^ dose is the first booster and the 5^th^ dose is the second booster. The dotted horizontal line is the responder cut-off of the NTCHIP^®^ assay for RBD-Wuhan (9.0 IU/mL). The dotted vertical line is the responder cut-off of the T-SPOT COVID assay (40 SFC/10^6^ PBMCs).

When cellular and humoral immune responses were compared between patients with IEI and controls at three different time points (**Figure 5**), control individuals displayed a more homogeneous response for both antibodies and the cellular response. In contrast, patients with IEI had a much more variable humoral immune response, and some did not even reach the cutoff level for the antibody response.

The results presented in **Figure 5** reveal two distinct subgroups of patients with IEI regarding their humoral response at the time points evaluated. At one month post-1st booster, patients with negative antibodies (below the threshold of 9.1 IU/mL) included one case of Hyper-IgM, two of CVID, one of XLA and one of Combined Immunodeficiency. Among those with a borderline response (antibody levels at threshold of 9.1 IU/mL), there were three patients with CVID, one with XLA, one with Hyper-IgM and one with Combined Immunodeficiency.

At one month post-2nd booster, one patient with CVID had negative antibodies (the same who had 24.4 IU/mL after the 1st booster). Among those with a borderline response (antibody levels at threshold of 9.1 IU/mL), there were four patients with CVID and two with XLA. Except for one XLA patient, all the other five patients were previously described at one month post-1st booster.

These patterns suggest heterogeneity in the humoral response, even among patients receiving similar vaccine regimens, with a potential impact of individual characteristics, such as the type and severity of IEI, on the ability to generate neutralizing antibodies and the presence of antibodies in the commercial immunoglobulin preparations administered to these patients.

## 4 Discussion

We compared the humoral and cellular immune responses after SARS-CoV-2 booster vaccination schedules in patients with IEI to those of healthy controls. Patients with IEI responded to a three-dose SARS-CoV-2 immunization with cellular immunity similar to that of controls. However, the IEI had a lower humoral response. Boosters (4th and 5th vaccine doses for IEI group and 3rd and 4th vaccine doses for control group) increased both humoral and cellular immunity. The evaluation of the immune response to SARS-CoV-2 in individuals with IEI is essential to understand vaccine effectiveness and to identify the role of different immune components in controlling the infection.

The interaction between SARS-CoV-2 and the human immune system underscores critical aspects of the host-pathogen interplay, particularly involving innate and adaptive immunity. Type I interferons are essential during the initial stages of infection, serving as a critical line of defense against SARS-CoV-2 (25). Adaptive immunity, orchestrated by T and B cells, plays a pivotal role in modulating disease severity and facilitating viral clearance (26–29). T cells are important for containing the spread of infection, while B cells contribute by producing high-quality antibodies through affinity maturation, somatic mutations, and class switching. These processes enhance the humoral response, culminating in the production of neutralizing antibodies capable of blocking viral entry (30).

The importance of these immune mechanisms is evident in the increased susceptibility of individuals with antibody deficiencies, such as CVID or congenital agammaglobulinemia, who face significantly higher risks of severe COVID-19 and hospitalization compared to the general population (31–37). T-cell responses play a crucial role in protecting patients with IEI from severe COVID-19 and death by compensating for impaired humoral responses and providing broad, cross-reactive immunity (7–10). Although patients with IEI may show diminished responses to immunization, the administration of SARS-CoV-2 vaccines was able to protect Brazilian patients. Recent Brazilian guidelines offer SARS-CoV-2 vaccines with more recent variants for patients with IEI every 6 months (38), similar to the CDC guidelines (39).

Delmonte, Castagnoli, and Notarangelo (2022) (13) suggested that COVID-19 vaccines are effective and safe in patients with IEI, but they underscored the need for additional studies to assess the duration and robustness of immune responses to COVID-19 vaccines in patients with IEI, with larger cohorts and longitudinal assessments for more informed conclusions on vaccine efficacy in this heterogeneous population. Currently, few studies have monitored the long-term immune response to COVID-19 in immunocompromised patients. In this study, we systematically monitored the humoral and cellular immune responses after five COVID-19 vaccinations for up to 22 months.

Several studies have highlighted the role of T-cell responses in mitigating the severity of COVID-19, particularly in individuals with compromised B-cell function. Patients with B-cell deficiencies, such as those receiving anti-CD20 therapy or those with CVID, exhibit elevated T-cell responses to SARS-CoV-2 infection and vaccination. These enhanced T-cell responses, especially within the CD8^+^ T-cell compartment, are associated with reduced odds of severe COVID-19 (8). The highest risk patients are those with antibodies to type I interferons such as patients with thymoma or autoimmune polyendocrine syndrome type 1 (APS-1). Additional patients with inherited defects in the type I interferon pathway have also been identified, cementing this pathway as critical for the defense against SARS-CoV-2 (40, 41). Moreover, patients with IEIs who received COVID-19 mRNA vaccines demonstrated an increase in the breadth of SARS-CoV-2-specific T-cell clonotypes, even in the absence of seroconversion. These findings suggest that T-cell responses can compensate for the lack of antibody production and provide protection against severe disease (9). Additionally, the presence of cross-reactive T cells from previous exposures to common cold coronaviruses has been associated with protection from symptomatic and fatal SARS-CoV-2 infections. These cross-reactive T cells can recognize conserved epitopes across different coronaviruses, contributing to a robust immune response (10).

Following COVID-19 immunization, both patients with IEI and controls with the first three doses of original strain of BNT162b2 (Pfizer-BioNTech) had samples collected starting in January 2022 during intense circulation of the highly transmissible Omicron variant, which had some immune escape from vaccines then available (42). According to Wang et al. (2023) (42), all current circulating SARS-CoV-2 variants show high antibody evasion levels, suggesting that this is the main reason for the high transmissibility of the virus. Notably, many positive results, both in terms of the cellular response (especially to Nucleocapsid) and in the humoral response in these subgroups, may be due to asymptomatic or untested SARS-CoV-2 infections, since two-thirds of both patients and controls have not received the CoronaVac vaccine, which contains Nucleocapsid in composition.

A slight and steady increase in antibody levels was observed in patients with IEI, possibly indicating an increase related to IGRT, which contrasts with the profile observed in controls. In the control group, antibodies initially declined, followed by a significant increase after booster doses or infections (**Figure 2**). Thus, immunological protection against severe COVID-19 in patients with IEI with predominantly humoral dysfunction likely depends on vaccine-induced cellular responses (7–10), as well as specific antibodies generated through immunoglobulin replacement therapies (43–46). Vaccination and passive immunity from IGRT may not always be sufficient for protection. Also, new SARS-CoV-2 variants may not be neutralized with the same effectiveness (47) with the XBB1.5 variant, highlighting the importance of booster doses with new vaccines as offered twice a year by the Brazilian Ministry of Health for immunocompromised individuals since May 2024 (38).

Studies with different vaccine platforms have also shown the importance of a third dose in increasing both humoral and cellular immunity in patients with IEI (48–54). There are presently six studies that have evaluated humoral and/or cellular response after the 4th dose: three with 33, 25 and 17 patients with CVID (47, 49, 55) and three with 30, 25 and 19 patients with different IEIs (24, 48, 51). A recent study reinforced our data on the importance of boosters in increasing the percentage of patients with IEI who do not respond to the initial doses of the immunization scheme but do so after the fourth dose (47). The booster effect is of importance in the IEI community. A waning enthusiasm for vaccination and limited ongoing public health efforts directed as COVID-19 requires that clinicians caring for patients with IEI continue to promote boosters.

Before the beginning of SARS-CoV-2 immunization in Brazil, the mortality of patients with IEI was twice that reported in the general population (31). A recent USA study with 823 patients with various IEIs who experienced COVID-19 showed that those who were immunized with at least one vaccine prior to infection had significantly lower rates of hospitalization and intensive care unit admission than nonvaccinated individuals did (56). However, as shown in a Swedish study, after receiving two COVID-19 vaccine doses, patients with IEI are still at greater risk of hospitalization than the general population (57). In the present study, after COVID-19 vaccination with four doses, no patients with IEI who became infected with SARS-CoV-2 developed severe disease. However, at earlier intervals with fewer vaccines administered, there were still hospitalizations in patients with IEI. The rationale of comparing different booster schedules between IEI and Control cohorts as we proposed is the same of observed for hepatitis B vaccine, in which immunocompromised individuals should receive an additional dose to ensure adequate protection against the virus (58), once the vaccination with a 4-dose scheme can be more effective than that 3-dose scheme (59).

This study had some limitations. We did not exclude participants with prior SARS-CoV-2 infection because of the potential impact on sample size. Additionally, it was not possible to evaluate the immune response in participants before the first COVID-19 vaccine dose. Also, we cannot exclude that asymptomatic and/or untested SARS-CoV-2 infections may have occurred. This study included a heterogenous group of patients and represents a real-life cohort with mixed vaccine types administered.

Nevertheless, there are strengths in our study. To our knowledge, this is the only study that has evaluated humoral responses to Spike, Nucleocapsid, Wuhan, Delta and Omicron BA.1, BA.2, and BA.5, in addition to the cellular immune response to Nucleocapsid and Spike, until one month after the 5th COVID-19 vaccine dose (second booster for immunocompromised patients). The high rate of COVID-19 in Brazil allowed for a crucial and robust analysis of COVID-19 hospitalization after vaccination.

In summary, our study demonstrated that patients with IEI exhibit a robust specific T-cell response to SARS-CoV-2 following vaccination, similar to that of healthy controls, but with a significantly lower humoral response. Booster doses (4th and 5th for the IEI group and 3rd and 4th for the control group) enhanced both humoral and cellular immunity. Furthermore, we observed a reduction in hospital admissions due to SARS-CoV-2 infection among vaccinated IEI patients, reinforcing the clinical relevance of our findings. These findings reinforce the importance of booster strategies to improve protection in these patients, who are at higher risk of severe disease and related complications from COVID-19. It is important to acknowledge that the immune response in individuals with IEI may be more heterogeneous and complex than in healthy populations due to the diversity of underlying immune defects.

Future research directions include long-term follow-up of immune responses in patients with IEI and investigation of the role of immunoglobulin replacement therapies in modulating vaccine immune responses. Data collection on responses to new variants of SARS-CoV-2 will also be essential to understand the impact of booster doses, as well as the role of T-cell responses in protection against emerging variants.

## Data Availability

The original contributions presented in the study are included in the article/**Supplementary Material**. Further inquiries can be directed to the corresponding authors.
