# Supplementary material for: Enhanced T-cell immunity and lower humoral responses following 5-dose SARS-CoV-2 vaccination in patients with inborn errors of immunity compared with healthy controls
Source: Front Immunol. 2025 Mar 6;16:1538453. doi: 10.3389/fimmu.2025.1538453 (PMC11922935; doi:10.3389/fimmu.2025.1538453)
Supplement: Supplementary file 1 [file Presentation1.pdf]

Supplementary Material

Enhanced T-cell immunity and lower humoral responses following 5-dose SARS-CoV-2 vaccination in patients with inborn errors of immunity compared with healthy controls

Frontiers in Immunology | Vaccines and Molecular Therapeutics

Vitor Gabriel Lopes da Silva[1*], Gabriela Justamante Händel Schmitz[2], Kathleen E. Sullivan[3], Júlia Barbate[1], Maria Izabel de Haro Azinar[1], Carolina Sanchez Aranda[1], Maria Isabel de Moraes-Pinto[1*]

[1]Universidade Federal de São Paulo, Escola Paulista de Medicina, Departamento de Pediatria, São Paulo, SP, Brazil.

[2]Universidade de São Paulo, São Paulo, SP, Brazil.

[3]The Children's Hospital of Philadelphia; Perelman School of Medicine, University of Pennsylvania, Philadelphia, PA, United States.

Correspondence | Corresponding Authors:

- Maria Isabel de Moraes-Pinto, MD, PhD, Laboratório de Pesquisas do Departamento de Pediatria, Universidade Federal de São Paulo, Rua Pedro de Toledo, 781, 9° andar, 04039030, Vila Clementino, São Paulo, Brazil. E-mail: m.isabelmp@gmail.com.
- Vitor Gabriel Lopes da Silva, PhD, Universidade Federal de São Paulo. E-mail: vitor.lopes@unifesp.br.

Keywords: COVID-19 vaccines, Booster, Inborn Errors of Immunity, Primary immunodeficiency disorders, SARS-CoV-2, Microarray, Immune response, ELISpot enzyme-linked immunospot.

Citation:
Lopes da Silva VG, Schmitz GJH, Sullivan KE, Barbate J, de Haro Azinar MI, Aranda CS and de Moraes-Pinto MI (2025). Enhanced T-cell immunity and lower humoral responses following 5-dose SARS-CoV-2 vaccination in patients with inborn errors of immunity compared with healthy controls. Front. Immunol. 16:1538453. doi: 10.3389/fimmu.2025.1538453

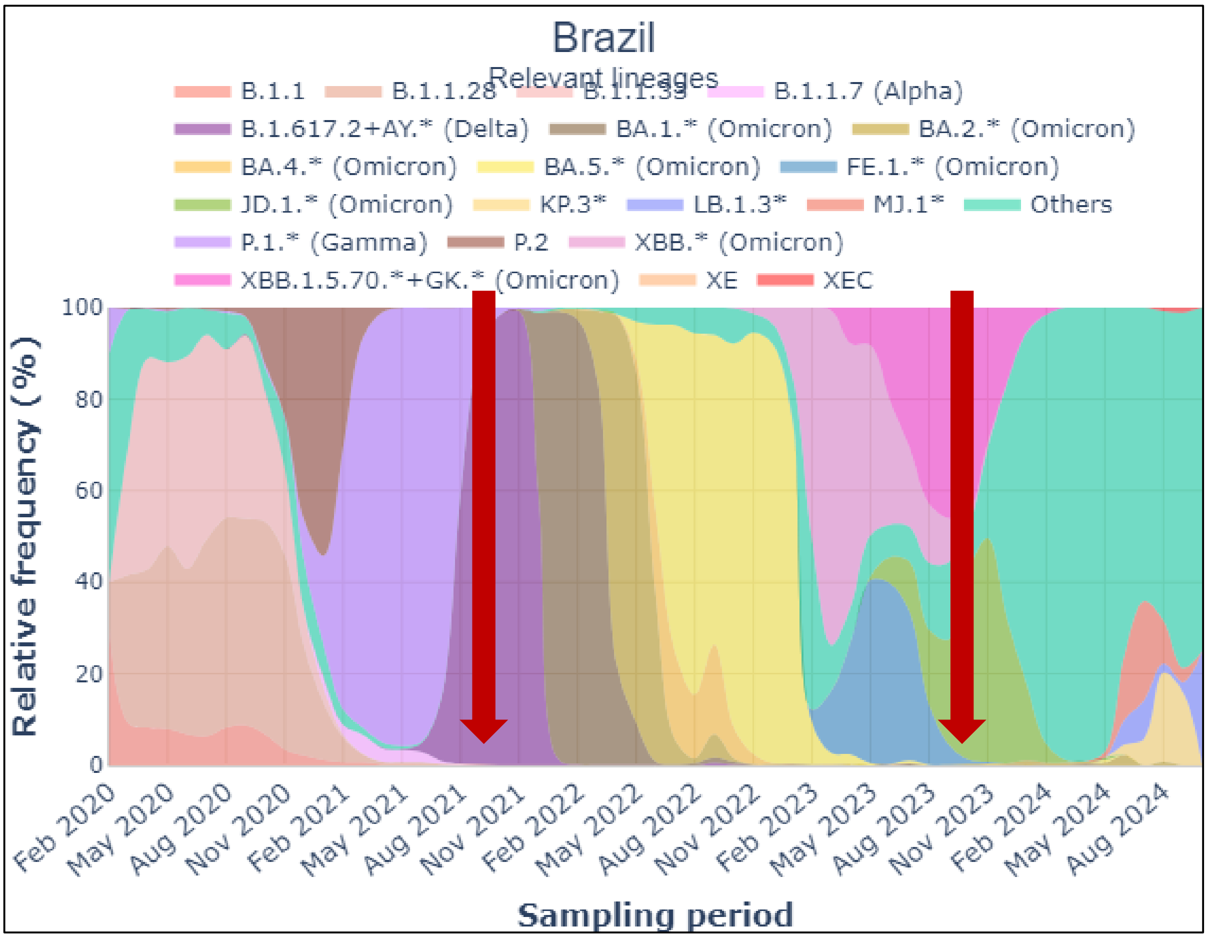

Supplementary Figure 1. **Relevant SARS-CoV-2 lineages in Brazil and study period.** Genomes generated from samples collected in Brazil and deposited on GISAID by the Fiocruz Genomic Network or other institutions are shown. Data available on https://www.genomahcov.fiocruz.br/dashboard-en/. Accessed on October 24, 2024. Study period is marked by **red** arrows.

METHODS

Humoral immune response of SARS-CoV-2 using NTChip® assay

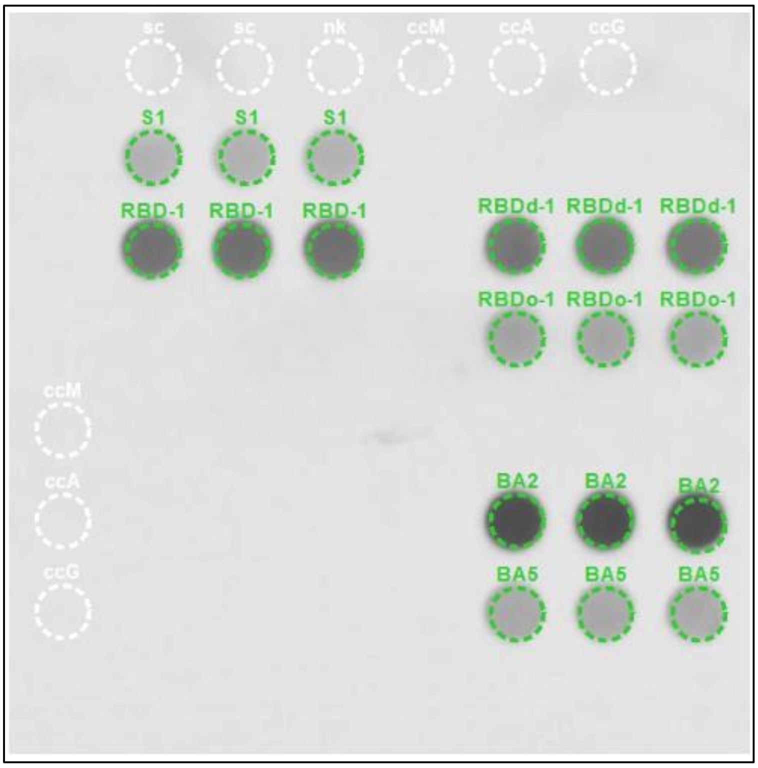

Supplementary Figure 2. **Picture of a negative control well from one plate of NTChip assay.** Results from the microarray readings were obtained after the first incubation for the detection of neutralizing antibodies against Spike, RBD-Wuhan, RBD-Delta, RBD-BA.1, RBD-BA.2, and RBD-BA.5. The intensity of the color in the triplicates of each antigens is inversely proportional to the quantity of neutralizing anti-RBD antibodies detected in the serum sample.

Cellular immune response of SARS-CoV-2 using ELISpot assay

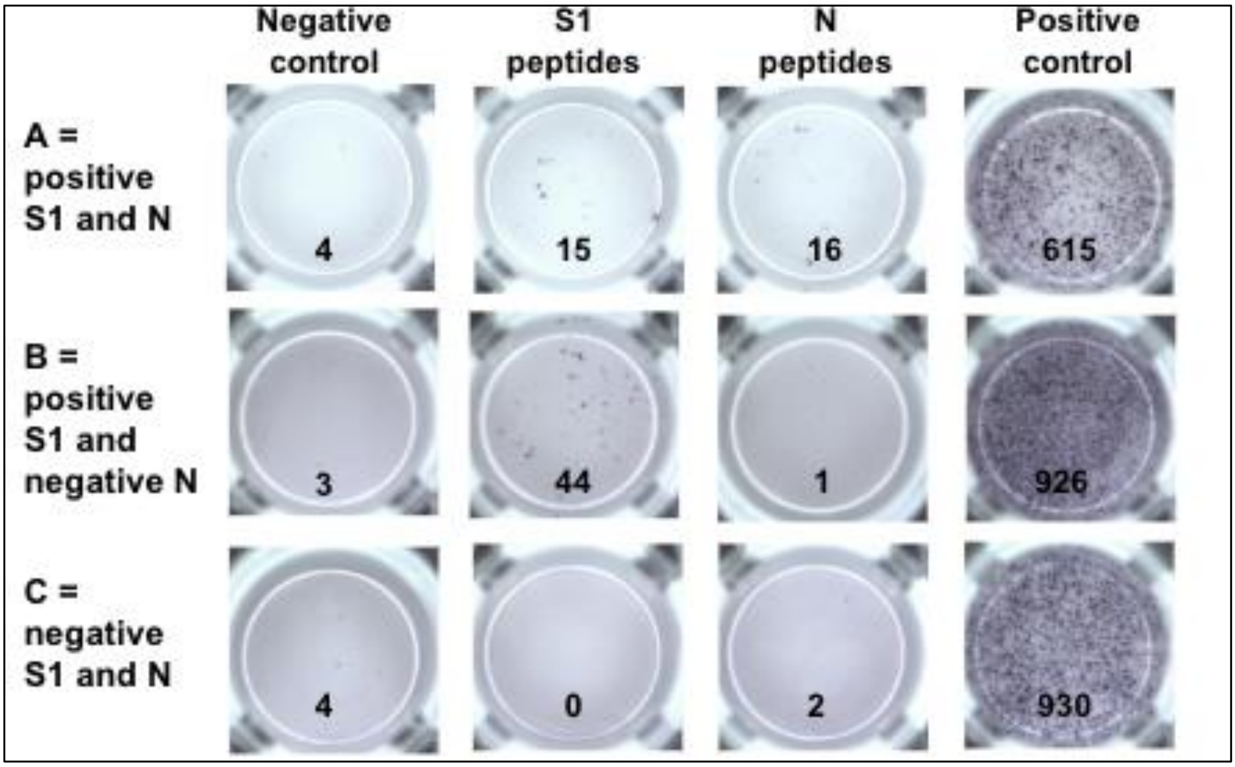

Supplementary Figure 3. **TSPOT.COVID results of three samples.** (A) positive results for Spike (S1) and Nucleocapsid (N), (B) positive only for Spike, and (C) negative for both antigens. The test is considered valid when the negative control has fewer than 10 spots and the positive control has more than 20 spots. The response to Spike or Nucleocapsid is considered positive when the number of spots minus the spots in the negative control is greater than or equal to 8 spots.

RESULTS

Table S1. Percentages of positive responses for neutralization of RBD-Wuhan and RBD-BA.1, for neutralizing antibodies in IU/mL anti-RBD-Wuhan and anti-RBD-BA.1, as well as anti-IgG Nucleocapsid antibodies in BAU/mL of SARS-CoV-2 following the booster schedules for IEI (4th and 5th doses) and Controls (3rd and 4th doses) of the COVID-19 vaccines evaluated by the NTChip assay (NIBSC 21/338).

	Time of evaluation			p		
	**1-month after 1st booster N (%)**	**6-months after 1st booster N (%)**	**1-month after 2nd booster N (%)**	**Group**	**Time**	**Group X Time**
**Wuhan[1] (Seropositivity for % Neutralization)**				-	-	-
Control	60/60 (100.0)	56/56 (100.0)	58/58 (100.0)			
IEI	40/49 (81.6)	46/49 (93.9)	36/42 (85.7)			
**BA.1 (Seropositivity for % Neutralization)**				**0.023**	0.279	0.392
Control	53/60 (88.3)	45/56 (80.4)	53/58 (91.4)			
IEI	31/49 (63.3)	32/49 (65.3)	29/42 (69.0)			
**Wuhan[2] (Seropositivity for IU/mL)**				-	-	-
Control	60/60 (100.0)	56/56 (100.0)	58/58 (100.0)			
IEI	45/49 (91.8)	46/49 (93.9)	41/42 (97.6)			
**BA.1 (Seropositivity for IU/mL)**				**0.033**	**<0.001**	0.363
Control	59/60 (98.3)	45/56 (80.4)	57/58 (98.3)			
IEI	41/49 (83.7)	32/49 (65.3)	39/42 (92.9)			
**Nucleocapsid (Seropositivity for BAU/mL)**				0.146	**<0.001**	0.219
Control	46/60 (76.7)	37/54 (68.5)	53/57 (93.0)			
IEI	38/49 (77.6)	23/48 (47.9)	38/42 (90.5)			

(-) Not estimable – all patients in the Control group showed positive responses in all assessments.
p – descriptive level of the logistic model with random effects. The **bold** value represents statistically significant results.
Results marked in pink indicate significantly higher values over time compared to those represented in blue. Results marked in orange indicate significantly higher values over time compared to those in blue, but lower than those in pink. Results with red font indicate values significantly higher than those represented in black font, among vertically distributed subgroups.
1: Time effect: p = 0.079. Test for the proportion of positive responses equal to 1 (100.0%) at all evaluation points: p = 0.001.
2: Time effect: p = 0.386. Test for the proportion of positive responses equal to 1 (100.0%) at all evaluation points: p = 0.061.

Table S2. Geometric Mean Titers (GMT) of neutralizing antibodies anti-RBD-Wuhan, anti-RBD-Delta, anti-RBD-BA.1, anti-RBD-BA.2, anti-RBD-BA.5 and anti-Nucleocapsid of SARS-CoV-2 following the booster schedules for IEI (4th and 5th doses) and Controls (3rd and 4th doses) of COVID-19 vaccines evaluated by the NTChip assay.

	**Time of evaluation**			**p**		
	**1-month after 1st booster GMT (95% CI)**	**6-months after 1st booster GMT (95% CI)**	**1-month after 2nd booster GMT (95% CI)**	**Group**	**Time**	**Group X Time**
**Wuhan**				**<0.001**	0.173	0.201
Control	298.2 (295.0 to 301.5)	286.6 (277.6 to 295.9)	296.4 (292.7 to 300.0)			
IEI	111.9 (72.1 to 173.7)	112.0 (74.2 to 169.1)	134.7 (88.9 to 204.0)			
**Delta**				**<0.001**	0.376	0.343
Control	263.6 (248.8 to 279.3)	262.2 (245.8 to 279.7)	267.7 (255.2 to 280.7)			
IEI	84.3 (49.5 to 143.6)	82.1 (48.8 to 138.3)	103.6 (60.3 to 178.3)			
**BA.1**				**0.016**	**<0.001**	0.078
Control	4,579.6 (3,422.7 to 6,127.6)	3,637.2 (2,558.8 to 5,170.0)	8,758.4 (6,736.5 to 11,387.3)			
IEI	2,668.8 (1,851.3 to 3,847.3)	2,909.9 (1,861.6 to 4,548.5)	4,277.6 (2,764.8 to 6,618.3)			
**BA.2**				**<0.001**	**<0.001**	**0.008**
Control	6,603.1 (5,159.3 to 8,451.1)	4,266.9 (3,124.3 to 5,827.4)	8,402.4 (6,513.8 to 10,838.5)			
IEI	1,975.0 (1,217.8 to 3,203.1)	2,419.0 (1,567.2 to 3,733.9)	3,002.1 (1,690.9 to 5,329.9)			
**BA.5**				**0.002**	**<0.001**	0.056
Control	4,547.3 (3,097.5 to 6,675.6)	4,094.7 (2,794.8 to 5,999.1)	8,657.4 (6,171.7 to 12,144.2)			
IEI	1,476.9 (795.2 to 2,743.1)	2,543.9 (1,444.4 to 4,480.3)	3,000.0 (1,489.3 to 6,043.2)			
**Nucleocapsid**				**0.004**	**<0.001**	**0.010**
Control	30.4 (23.1 to 39.9)	49.5 (35.0 to 69.8)	87.8 (68.1 to 113.1)			
IEI	30.7 (22.6 to 41.7)	32.4 (21.8 to 48.3)	42.2 (30.3 to 58.9)			

p – descriptive level of the linear model with random effects. The **bold** value represents statistically significant results. Results marked in pink indicate significantly higher values over time compared to those represented in blue.

Table S3. GMT of neutralizing antibodies anti-RBD-Wuhan, anti-RBD-Delta, anti-RBD-BA.1, anti-RBD-BA.2, anti-RBD-BA.5 and anti-Nucleocapsid of SARS-CoV-2 following the booster schedules for IEI and Controls of COVID-19 vaccines, separated by the vaccines received in the first two doses, evaluated by the NTChip assay.

	Time of evaluation			p		
	**1-month after 1st booster GMT (95% CI)**	**6-months after 1st booster GMT (95% CI)**	**1-month after 2nd booster GMT (95% CI)**	**Group**	**Time**	**Group x Time**
**Wuhan**				**<0.001**	0.185	0.233
Control - ChAdOx1 nCoV-19	299.6 (298.2 to 301.0)	282.5 (263.7 to 302.6)	299.1 (297.4 to 300.7)			
Control - CoronaVac	294.3 (284.7 to 304.2)	294.2 (284.8 to 303.9)	297.9 (295.8 to 300.1)			
Control - BNT162b2	300.8 (300.8 to 300.8)	282.6 (263.3 to 303.3)	292.0 (281.1 to 303.4)			
IEI - ChAdOx1 nCoV-19	139.0 (51.0 to 378.8)	146.3 (49.8 to 430.1)	116.2 (33.3 to 405.8)			
IEI - CoronaVac	103.8 (52.0 to 207.2)	114.1 (62.8 to 207.5)	147.3 (87.5 to 247.9)			
IEI - BNT162b2	111.3 (48.8 to 254.0)	92.4 (40.2 to 212.2)	130.0 (51.9 to 325.7)			
**Delta**				**<0.001**	0.243	0.236
Control - ChAdOx1 nCoV-19	260.3 (233.9 to 289.8)	252.1 (223.6 to 284.3)	272.8 (253.0 to 294.1)			
Control - CoronaVac	266.7 (240.1 to 296.1)	286.0 (268.0 to 305.3)	263.0 (241.6 to 286.2)			
Control - BNT162b2	263.9 (237.0 to 293.9)	247.2 (211.3 to 289.2)	267.6 (242.0 to 295.9)			
IEI - ChAdOx1 nCoV-19	108.6 (33.6 to 351.2)	143.5 (67.1 to 306.9)	95.4 (20.6 to 441.5)			
IEI - CoronaVac	79.9 (35.2 to 181.8)	90.4 (42.8 to 191.0)	119.7 (61.8 to 231.7)			
IEI - BNT162b2	79.2 (27.6 to 227.1)	50.1 (15.2 to 164.8)	88.0 (24.5 to 316.1)			
**BA.1**				0.276	**<0.001**	0.419
Control - ChAdOx1 nCoV-19	3,305.5 (1,964.1 to 5,563.1)	3,407.1 (1,731.4 to 6,704.9)	7,999.4 (5,118.6 to 12,501.5)			
Control - CoronaVac	4,591.3 (2,661.5 to 7,920.3)	2,760.8 (1,531.9 to 4,975.4)	8,254.1 (5,016.9 to 13,580.1)			
Control - BNT162b2	6,328.7 (3,848.9 to 10,406.3)	5,411.7 (2,847.5 to 10,285.0)	10,207.1 (6,206.2 to 16,787.4)			
IEI - ChAdOx1 nCoV-19	2,905.1 (1,311.3 to 6,436.0)	2,983.5 (957.3 to 9,298.3)	4,511.7 (1,577.5 to 12,903.2)			
IEI - CoronaVac	2,233.7 (1,292.2 to 3,861.3)	2,846.5 (1,507.4 to 5,375.3)	3,982.6 (1,980.6 to 8,008.2)			
IEI - BNT162b2	3,412.0 (1,620.7 to 7,183.4)	2,973.8 (1,144.3 to 7,728.6)	4,601.8 (1,995.3 to 10,613.3)			
**BA.2**				**0.001**	**<0.001**	**0.034**
Control - ChAdOx1 nCoV-19	4,774.9 (2,941.0 to 7,752.3)	3,649.7 (1,999.9 to 6,660.8)	8,068.0 (5,427.3 to 11,993.7)			
Control - CoronaVac	7,431.3 (4,767.9 to 11,582.6)	3,219.0 (2,031.4 to 5,101.0)	8,092.1 (5,107.4 to 12,821.1)			
Control - BNT162b2	8,113.7 (5,497.5 to 11,975.0)	7,078.3 (3,891.2 to 12,876.1)	9,104.2 (5,298.3 to 15,643.8)			
IEI - ChAdOx1 nCoV-19	1,945.6 (550.5 to 6,876.2)	3,037.3 (938.9 to 9,824.9)	3,038.9 (672.0 to 13,743.0)			
IEI - CoronaVac	2,168.4 (1,101.8 to 4,267.2)	2,103.4 (1,148.0 to 3,853.9)	3,364.9 (1,484.2 to 7,629.0)			
IEI - BNT162b2	1,705.6 (603.0 to 4,824.4)	2,663.9 (1,066.9 to 6,651.6)	2,497.6 (732.3 to 8,518.7)			
**BA.5**				0.121	**0.001**	0.332
Control - ChAdOx1 nCoV-19	3,407.7 (1,614.8 to 7,191.1)	3,914.0 (1,888.6 to 8,111.6)	7,925.5 (4,466.0 to 14,065.0)			
Control - CoronaVac	5,202.7 (2,815.2 to 9,615.0)	2,906.5 (1,681.5 to 5,023.8)	8,305.9 (4,695.6 to 14,692.2)			
Control - BNT162b2	5,303.5 (2,545.8 to 11,048.3)	6,445.2 (2,892.7 to 14,360.5)	9,878.6 (4,806.5 to 20,302.8)			
IEI - ChAdOx1 nCoV-19	1,757.2 (356.7 to 8,657.7)	3,464.3 (558.7 to 21,479.7)	2,870.3 (485.4 to 16,973.7)			
IEI - CoronaVac	1,321.9 (529.2 to 3,302.0)	2,455.7 (1,165.2 to 5,175.5)	3,683.5 (1,371.1 to 9,895.9)			
IEI - BNT162b2	1,600.8 (464.6 to 5,516.3)	2,241.8 (699.8 to 7,181.2)	2,255.6 (484.8 to 10,494.5)			
**Nucleocapsid**				**0.001**	**<0.001**	**0.012**
Control - ChAdOx1 nCoV-19	19.4 (13.7 to 27.5)	25.2 (14.1 to 45.0)	75.6 (46.3 to 123.7)			
Control - CoronaVac	30.8 (18.3 to 51.9)	66.8 (36.2 to 123.5)	110.4 (71.4 to 170.7)			
Control - BNT162b2	46.7 (27.7 to 78.8)	77.1 (44.5 to 133.5)	81.0 (51.3 to 127.8)			
IEI - ChAdOx1 nCoV-19	45.3 (21.1 to 97.1)	54.0 (18.0 to 161.6)	48.4 (22.0 to 106.1)			
IEI - CoronaVac	32.1 (19.1 to 53.8)	38.1 (21.2 to 68.4)	42.9 (24.7 to 74.5)			
IEI - BNT162b2	22.6 (15.7 to 32.4)	18.9 (9.6 to 37.4)	37.5 (20.8 to 67.6)			

p – descriptive level of the linear model with random effects. The bold value represents statistically significant results.
Results marked in pink indicate significantly higher values over time compared to those represented in blue.

Table S4. Quantitative IFN-gamma cellular response in SFC (spot-forming cells) per million PBMCs (peripheral blood mononuclear cells) in IEI and Controls for specific antigens (S1 subunit of the Spike protein and Nucleocapsid) of SARS-CoV-2 following the booster schedules of COVID-19 vaccines.

	Time of evaluation			p		
	1-month after 1st booster Mean ± SD	6-months after 1st booster Mean ± SD	1-month after 2nd booster Mean ± SD	Group	Time	Group x Time
**Nucleocapsid**				0.180	**0.017**	0.763
Control	64.1 ± 156.2	75.3 ± 88.9	133.9 ± 143.8			
IEI	126.4 ± 273.5	166.4 ± 208.0	185.4 ± 345.1			
**Spike**				**0.002**	0.445	0.695
Control	128.6 ± 183.4	108.4 ± 196.5	149.8 ± 196.7			
IEI	395.6 ± 644.3	321.4 ± 532.2	405.2 ± 496.9			

p – descriptive level of the linear model with random effects. The **bold** value represents statistically significant results.
Results marked in pink indicate significantly higher values over time compared to those represented in blue.

Table S5. Quantitative IFN-gamma cellular response in SFC (spot-forming cells) per million PBMCs (peripheral blood mononuclear cells) for specific antigens (S1 subunit of the Spike protein and Nucleocapsid) of SARS-CoV-2 following the booster schedules of COVID-19 vaccines for IEI and Controls, separated by the vaccines received in the first two doses.

	Time of evaluation						p		
	1-month after 1st booster Mean ± SD (A)	6-months after 1st booster Mean ± SD (B)	1-month after 2nd booster Mean ± SD (C)	N_A	N_B	N_C	Group	Time	Group x Time
**Nucleocapsid**							0.443	0.052	0.554
Control - ChAdOx1 nCoV-19	26.7 ± 92.2	54.8 ± 74.0	152.6 ± 137.0	20	19	19			
Control - CoronaVac	51.3 ± 68.9	70.4 ± 101.1	151.3 ± 139.8	20	20	20			
Control - BNT162b2	114.4 ± 241.2	107.8 ± 85.4	97.0 ± 154.7	20	15	19			
IEI - ChAdOx1 nCoV-19	69.7 ± 67.0	121.8 ± 144.1	105.9 ± 240.6	9	9	9			
IEI - CoronaVac	116.5 ± 135.6	182.0 ± 213.2	201.1 ± 212.8	25	24	19			
IEI - BNT162b2	176.9 ± 466.3	168.3 ± 238.9	217.6 ± 533.0	15	15	13			
**Spike**							0.063	0.462	0.554
Control - ChAdOx1 nCoV-19	109.8 ± 160.7	92.3 ± 144.5	152.5 ± 238.2	20	19	19			
Control - CoronaVac	57.1 ± 121.0	63.9 ± 67.4	171.8 ± 210.5	20	20	20			
Control - BNT162b2	218.8 ± 222.8	188.3 ± 321.1	124.1 ± 133.0	20	15	19			
IEI - ChAdOx1 nCoV-19	461.7 ± 935.3	452.0 ± 972.4	466.4 ± 754.0	9	9	9			
IEI - CoronaVac	372.7 ± 456.0	264.7 ± 323.1	450.9 ± 450.2	25	24	19			
IEI - BNT162b2	393.9 ± 750.3	333.7 ± 471.5	296.0 ± 347.8	15	15	13			

p – descriptive level of the linear model with random effects.
